# The relationship between tumour glutathione concentration, glutathione S-transferase isoenzyme expression and response to single agent carboplatin in epithelial ovarian cancer patients.

**DOI:** 10.1038/bjc.1996.384

**Published:** 1996-08

**Authors:** S. Ghazal-Aswad, L. Hogarth, A. G. Hall, M. George, D. P. Sinha, M. Lind, A. H. Calvert, J. P. Sunter, D. R. Newell

**Affiliations:** Cancer Research Unit, University of Newcastle-upon-Tyne, UK.

## Abstract

There is evidence to suggest that glutathione (GSH) and glutathione-S-transferases (GST) are important factors in determining sensitivity to cytotoxic drugs in vitro and in preclinical in vivo model systems. To define the relationship between tumour GSH concentration, GST isoenzyme expression and response to carboplatin in epithelial ovarian cancer (EOC), tumour samples from 39 patients with assessable disease after primary surgery were analyzed for GSH content and GST expression. Response was assessed after completing six courses of single agent carboplatin therapy. GSH was measured by high performance liquid chromatography (HPLC) in fresh tumour samples taken at primary laparatomy. GST isoenzyme expression was assessed by immunohistochemistry of fixed tumour material using antibodies specific for pi, alpha and mu classes. GST isoenzyme expression was defined as positive if the staining intensity was strong and more than 10% of tumour cells were involved. The mean GSH concentrations were: 8351 +/- 4496, 7211 +/- 5026, 6559 +/- 4573 and 3758 +/- 1885 (nmol g-1 tissue dry weight mean +/- s.d.) for tumours from patients who subsequently achieved a complete response (CR, n = 18), partial response (PR, n = 10) or who had static disease (SD, n = 7) or progressive disease (PD, n = 4) respectively. There was no relationship between GSH concentration and response (ANOVA, P = 0.32). There were also no relationship between GST isoenzyme expression and response (P Fisher's exact test 0.51-0.55 and chi-squared test 0.98-0.99). In conclusion, there was no association between the concentration of GSH or expression of GST isoenzymes and response to single agent carboplatin in primary previously untreated EOC.


					
Bridsh Journal of Cancer (1996) 74, 468-473
? 1996 Stockton Press All rights reserved 0007-0920/96 $12.00

The relationship between tumour glutathione concentration, glutathione S-
transferase isoenzyme expression and response to single agent carboplatin
in epithelial ovarian cancer patients

S Ghazal-Aswad1, L Hogarth2, AG Hall2, M George3, DP Sinha3, M Lind', AH Calvert',
JP Sunter4 and DR Newell'

'Cancer Research Unit and 2Leukaemia Research Fund Unit, University of Newcastle-upon-Tyne, Newcastle-upon-Tyne NE2 4HH;
3Regional Department of Gynaecological Oncology and 4Department of Pathology, Queen Elizabeth Hospital, Gateshead NE9 6SX
UK.

Summary There is evidence to suggest that glutathione (GSH) and glutathione-S-transferases (GST) are
important factors in determining sensitivity to cytotoxic drugs in vitro and in precinical in vivo model systems.
To define the relationship between tumour GSH concentration, GST isoenzyme expression and response to
carboplatin in epithelial ovarian cancer (EOC), tumour samples from 39 patients with assessable disease after
primary surgery were analysed for GSH content and GST expression. Response was assessed after completing
six courses of single agent carboplatin therapy. GSH was measured by high performance liquid
chromatography (HPLC) in fresh tumour samples taken at primary laparotomy. GST isoenzyme expression
was assessed by immunohistochemistry of fixed tumour material using antibodies specific for xr, a and p classes.
GST isoenzyme expression was defined as positive if the staining intensity was strong and more than 10% of
tumour cells were involved. The mean GSH concentrations were: 8351+4496, 7211+5026, 6559+4573 and
3758 + 1885 (nmol g-I tissue dry weight mean + s.d.) for tumours from patients who subsequently achieved a
complete response (CR, n =18), partial response (PR, n = 10) or who had static disease (SD, n = 7) or
progressive disease (PD, n =4) respectively. There was no relationship between GSH concentration and
response (ANOVA, P = 0.32). There were also no relationship between GST isoenzyme expression and response
(P Fisher's exact test 0.51-0.55 and chi-squared test 0.98-0.99). In conclusion, there was no association
between the concentration of GSH or expression of GST isoenzymes and response to single agent carboplatin
in primary previously untreated EOC.

Keywords: glutathione; glutathione S-transferase; carboplatin; resistance; ovarian cancer

Single agent carboplatin therapy after primary surgery has
been standard treatment in the Northern Region (UK) for
patients with advanced epithelial ovarian cancer (EOC stages
Ic-IV). A meta-analysis indicates that 56% of patients with
advanced ovarian cancer will respond to single agent
carboplatin as first-line therapy, suggesting that the
remainder have tumours which are intrinsically resistant
(Rozencwig et al., 1990). Of the patients who do respond to
first-line carboplatin, some 60% will subsequently relapse and
hence acquired resistance is also a significant problem.
Resistance to platinum complexes is multifactorial and
includes: decreased accumulation of drug, increased intracel-
lular detoxification, which can involve glutathione (GSH),
glutathione S-transferases (GSTs) and metallothioneins,
increased DNA repair and increased tolerance of unrepaired
DNA damage. Notably, several mechanisms may operate
concomitantly (De Graeff et al., 1988). Furthermore,
essentially all of the data on resistance to platinum
compounds are derived from cell lines or experimental
animal systems, and it is uncertain whether the mechanisms
defined in these model systems are relevant to the clinical
setting.

Of all the mechanisms implicated in preclinical studies,
GSH and its metabolism is the one most commonly identified
in resistance to cisplatin and carboplatin. GSH is tripeptide,
gamma-glutamylcysteinylglycine, and is the most abundant
non-protein thiol found in the cell. GSH is present in
millimolar concentrations and is found throughout the cell,
with the bulk in the cytoplasm; subcellular particles,. such as

the nucleus and mitochondria, having smaller amounts
(Biaglow and Tuttle, 1993). GSH plays a role in the
detoxification and repair of cell injury induced by a wide
range of toxic agents which include cytotoxic drugs, radiation
and hyperthermia (Biaglow and Tuttle, 1993). GSH functions
primarily through nucleophilic thioether formation and
peroxidation/reduction reactions. The exact mechanism by
which GSH decreases platinum complex cytotoxicity is not
well established; but the following possibilities have been
proposed (reviewed by De Graeff et al., 1988): alteration of
platinum complex transport, drug inactivation through the
formation of an inactive GSH - Pt complex, decreased
binding of platinum to DNA and increased DNA repair.
More recently data have been published which indicate that
cisplatin may be extruded from cells as a glutathione
conjugate, and that increased expression of the GS-X pump
may be associated with drug resistance (Ishikawa and Ali-
Osman, 1993; Ishikawa et al., 1994).

The cytosolic GSTs are a multigene family of enzymes that
exist as homo- and hetero-dimers each of which have a
molecular weight of between 21 and 28 kDa (Mantle, 1990;
Gulick and Fahl, 1995). Four different classes of enzyme have
been described, a, p, ir and 0 which differ in immunor-
eactivity. Monomers of one class will not dimerise with those
of another. Within each class there may be several different
isoforms which share amino acid homology but may differ
significantly in substrate specificity. GSTs have been shown
to detoxify a wide range of xenobiotics (reviewed by Ciaccio
and Tew, 1993), including cytotoxic drugs, through both the
catalysis of glutathione conjugate formation (Hall et al.,
1994a; Bolton et al., 1991) and by sequestration (Meyer et al.,
1992).

Although there is evidence from studies of both cell lines
and clinical material that increased expression of GSTs and
raised levels of GSH may be associated with resistance to
cytotoxic drugs, the relationship between response and

Correspondence: DR Newell, Cancer Research Unit, Medical School,
University of Newcastle-upon-Tyne, Framlington Place, Newcastle-
upon-Tyne, NE2 4HH, UK

Received 27 February 1996; revised 27 February 1996; accepted 29
February 1996

GST isoenzyme expression and response to carboplatin
S Ghazal-Aswad et al !

469

expression in patients remains unclear. The aim of this study
was to relate response to single-agent carboplatin to tumour
levels of GSH and GST in EOC.

Materials and methods

Patients and tumour samples

EOC tumour samples were collected from 48 patients. Of
these, only 39 patients were included in the current analysis.
The remainder either had no residual disease after primary
laparotomy and hence were non-assessable for response to
chemotherapy (six patients) or GSH concentrations that
varied by more than 2-fold between different tumour pieces
(three patients). Samples (median = 1 and range 1-5 samples
from each patient) were collected immediately after primary
laparotomy, stored in liquid nitrogen within 10 min and
analysed in batches. The histology of the 39 tumour samples
analysed was reported as follows: adenocarcinoma in 12 cases
(1 moderately and 11 poorly differentiated), serous cystade-
nocarcinoma in 12 (one well, seven moderately and four
poorly differentiated), clear cell carcinoma in five (two well,
two moderately and one poorly differentiated), endometroid
carcinoma in seven (two well, three moderately and two
poorly differentiated) and mucinous cystadenocarcinoma in
three cases (one well, one moderately and one poorly
differentiated). There was no significant trend for the
relationship between tumour grade and response (chi-
squared test P= 0.87) and too few samples to study the
relationship between response and histological subtype.

Chemotherapy

All patients were treated with carboplatin as a single agent
and the dose was calculated either according to the surface
area (i.e. 400 mg m-2 in 13 patients) or according to renal
function with a target area under the plasma concentration vs
time curve of 7 mg ml-1 min-m (Calvert et al., 1989; in 26
patients). Response was assessed after completing six courses
of single-agent carboplatin and classified as a response
(complete or partial) or non-response (static or progressive
disease) according to standard criteria (UICC).

Chemicals

Chemicals were obtained from Sigma Chemical Co. Ltd.
(Poole, Dorset, UK), Aldrich Chemical Co. (Gillingham,
Dorset, UK) and BDH Chemical Co. (Poole, Dorset, UK).

Sample preparation for GSH measurements

Tumour tissue was removed from storage in liquid nitrogen
and cut while frozen into two sections. The first section was
weighed wet and then dried in a heating block at 50?C until
the weight was stable. The dry weight was recorded and the
wet weight to dry weight ratio was calculated. The second
tissue section was ground in liquid nitrogen using a pestle
and mortar. Once ground, the fragments were collected into a
cooled Teflon-glass homogeniser and homogenised in 25
volumes of 6.5% (w/v) TCA. Three 450 ,ul samples of the
homogenate were removed. An aliquot of 50 ,l 0.5 mM GSH
was added to the first of these aliquots and 50 ,l 1.0 mM
GSH to the second. An addition 50 MI of 6.5% (w/v) TCA
was added to the third aliquot. After adding the GSH
solutions, the samples were mixed and centrifuged (10 000 g,
12 min, 4C) using a Biofuge 15 (Heraeus Sepatech,
Germany). An aliquot of 75 ,il of 1 M potassium hydrogen
phosphate was mixed with 75 jl of the supernatant, 150 ,ul of
monobromobimane (mBrB) reagent was added (see below)
and, after gentle mixing, the sample incubated at room
temperature for 5 min in the dark. The reaction was stopped
with 15 ,ul of 100% (w/v) TCA and the samples were kept at
-80?C until analysed by HPLC within 2 weeks of
preparation.

High performance liquid chromatography measurement of GSH
in tumour samples

The method used was based upon the use of mBrB and is a
modification of the one described by Cotgreave and Moldeus
(1986). This method results in the maximal recovery of both
free and low molecular weight thiols from the tissue and, by
virtue of the HPLC separation used, gives specific detection
of GSH.

Monobromobimane reagent and standard curve prepara-
tion The reagent used was 8 mM monobromobimane
(Calbiochem, Nottingham, UK) in 50 mM N-ethylmorpho-
line (NEM). To prepare 25 ml of the mBrB reagent, 158 pl
NEM was added to 23 ml deionised water in a 30 ml glass
tube. MBrB (54.25 mg) was thoroughly dissolved in a
minimum amount of acetonitrile, the solutions of NEM
and mBrB were then mixed, and pH adjusted to 8.0, using
1 M hydrochloric acid, and then made up to 25 ml in a
volumetric flask with deionised water.

For preparation of the GSH standard curve, eight different
standard solutions were prepared (2.5, 5, 10, 20, 40, 60, 80
and 100 /iM of GSH). GSH (64.53 mg) was dissolved in
50 ml of phosphate-buffered saline (PBS), giving a concen-
tration of 4 mM GSH. A 0.25 ml aliquot of the solution was
diluted up to 10 ml in a volumetric flask, giving a total
concentration of 100 ,iM of GSH which was diluted serially
to give the concentrations indicated above. Fifty ,l of 30 mM
dithiothreitol (DTT), made up fresh every day in PBS, was
added to 1 ml of each of the above standard solutions and
the standards were then left for 30 min at room temperature.
MBrB reagent (130 pl) was mixed with 130 Ml of each of the
DTT-treated GSH standards and left in the dark for 5 min,
after which 13 pl of 100% (w/v) TCA was added. These final
solutions were analysed by HPLC without any further
dilution.

To assess the reproducibility of the assay, two different
solutions of GSH (5 and 80 /UM in PBS) were prepared. These
quality control standards were aliquoted and stored at
- 80?C. An aliquot of each concentration was analysed
with each assay.

HPLC apparatus Analysis of GSH was performed using a
Waters model 625 low-pressure mixing gradient HPLC
system fitted with a model 470 fluorescence detector
(Waters, Watford, UK). Samples were injected using a
Waters model 712 Wisp auto-injector and the results
analysed using Millipore Maxima chromatography software.

HPLC conditions The GSH mBrB adduct was analysed
using a S /im octadodecylsilica (ODS) reverse-phase column
(4.6 mm inside diameter x 15 cm length; 4M15115, Jones
Chromatography, Hengoed, UK). The solvent flow rate was
1 ml min-1, giving a back pressure of less than 2000 p.s.i.
The GSH peak (retention time 11.1 min) was measured by a
fluorescence detector (excitation 394 nm, emission 480 nm).
Chromatography was performed using a gradient of 10%
(v/v) acetonitrile, 0.25% (v/v) acetic acid in water against
75% (v/v) acetonitrile, 0.25% (v/v) acetic acid. Table I gives
the gradient conditions used for the HPLC separation of the
GSH mBrB adduct.

Calculation of final GSH concentration per gram of tissue dry
weight In this method, maximum efforts were made to
minimise and correct for any loss of GSH during the process
of the tissue collection or processing. The samples were
collected in less than 10 min after removal of the tumour
from the pelvis and selection of the tumour samples was
performed by an experienced pathologist (JPS). The tumour
tissue was handled and ground in liquid nitrogen before
homogenisation with 6.5% TCA. A method of additions
analysis was used, i.e. the addition of 0, 0.5 and 1.0 mM GSH
to each tumour sample homogenate, so that recovery of GSH
could be calculated for each individual sample and any loss

GST isoenzyme expression and response to carboplatin

S Ghazal-Aswad et at
470

Table I Gradient conditions used for the HPLC measurement of

glutathione

Phase        Time      Flow       % A       % B       % C
1             0.00      1.00       80        10        10
2             5.00      1.00       80        10        10
3            13.00      1.00       15        75        10
4            17.00      1.00       15        75        10
5            19.00      1.00       80        10        10

A, water; B, acetonitrile 2.5% (v/v); C, acetic acid 0.25% (v/v).

of GSH during processing compensated for. Figure 1 shows
the result for the method of addition in one patient sample.
The GSH concentration is given by the extrapolated x-axis
intercept. The recovery is given by the slope of the line which
was fitted by unweighed linear regression analysis (r2>0.99 in
all cases). When possible (18 patients) more than one sample
(2-5) was collected from different locations within the
tumour. After measurement of GSH concentration in each
sample separately, the mean value for all of the samples was
used as the GSH concentration value for the patient, unless
there was wide variation (>2-fold), in which case the patient
was considered inevaluable for GSH determination. The
original GSH content of the tumour tissue was expressed per
gram of tissue dry weight.

Immunohistochemical detection of glutathione-S-transferase
expression

The glutathione S-transferase (GST) isoenzyme distribution
was determined using three rabbit polyclonal antibodies
raised against the human 7t, a and ,u forms of the enzyme
(Hall et al., 1990). The use of polyclonal antibodies to detect
different GST isoenzymes has been described previously
(Hayes et al., 1989; Harrison et al., 1989; Murphy et al.,
1992). The principles of the methods of immunohistochemical
staining have been described by Burn (1979), and for GST
assessment in particular, by Randall et al. (1990).

Paraffin wax sections (4 gm thickness) were cut from the
tissue blocks and mounted on slides. The sections were dried,
dewaxed and rehydrated. After washing with water, the
sections were washed in Tris-buffered saline (TBS) (150 mM
sodium chloride, 5 mM Tris, pH 7.9 with 1 M hydrochloric
acid). The excess TBS was wiped and the sections were
incubated in normal swine serum (NSS) (Dako, High
Wycombe, Bucks, UK) for 10 min. The excess NSS was
removed and the primary anti-GST antibody was added at a
dilution of 1:400 in NSS. After overnight incubation at 4?C
the sections were rinsed with TBS. The excess TBS was wiped
and the sections were incubated in biotinylated anti-rabbit
secondary antibody (Dako) at a dilution of 1:20 in TBS.
Following incubation for 30 min at room temperature, the
sections were washed with TBS and then incubated in
streptavidin-biotin horseradish peroxidase (Dako) (1:200 in
NSS) solution for 30 min. The sections were washed and
immersed in TBS for 5 min. After wiping the slides, the
peroxidase reaction was developed using 3,3',-diaminobenzi-
dine tetrahydrochloride solution (DAB) for 10 min (80 mg
DAB in 100 ml TBS containing 0.086 mg imidazole). The
sections were washed with distilled water and treated with
copper sulphate enhancer for 1 min, thoroughly washed in
water and counter-stained with haematoxylin and mounted.

Tumour slides were stained in batches (10- 12 each). In
each batch, and to control the quality of the staining, positive
control slides were added to the batch (breast tumour for 7t,
kidney for a and positive liver control for y GSTs; supplied
by Novocastra Laboratories Ltd, Newcastle-upon-Tyne,
UK).

Scoring was performed jointly (JPS and SGA) and the
tissue sections selected for evaluation were those that only
contained malignant changes as determined by an experi-

60

4-c
0)
.-,5
0
I 'o
0 a
C,)

Q

0.

E

-

50
40
30
20
10

n

I                         I                        I            .            I            .            I           .            I            .            I

v

-20   -10    0     10    20    30    40

GSH added (nmol per 0.018 g wet weight)

50

Figure 1 Calculation of GSH concentration in tumour samples
by the method of standard additions (r2 = 0.999; recovery = 83%).

enced gynaecological cancer pathologist (JPS). Each GST
subgroup was scored separately. Tumour staining was scored
positive when the intensity was positive and > 10% of
tumour cells were involved, the 10% limit being selected to
avoid false positives. Scoring was performed without a
knowledge of the response status of the patient.

Statistical analyses

The relationship between GSH concentration and response
was studied statistically by analysis of variance. The
relationship between GST isoenzyme expression and re-
sponse to carboplatin was analysed according to both
Fisher's exact and chi-squared tests.

Ethical committee approval

A copy of the study protocol was submitted to the regional
ethics committee and approval was obtained before
commencement of the study.

Results

Tumour glutathione concentration in relationship to response to
carboplatin

GSH concentrations were measured and analysed in tumour
samples from 39 patients (78 different pieces). The recovery
of GSH varied between 62 and 111 %, and the mean recovery
rate was 93%. Figure 2 represents the individual values for
GSH concentrations in the tumours of 39 patients and their
relationship to response to subsequent carboplatin therapy.
Each point represents one sample or the mean of 2- 5
samples from the same point. The GSH values in the tumour
samples were: 8351+4496, 7211+5026, 6559+4573 and
3758+ 1885 (nmol g-' tissue dry weight, mean+s.d.) for
complete responders (CR, n= 18), partial responders (PR,
n = 18) and patients with static disease (SD, n = 7) or
progressive disease (PD, n =4) respectively. These mean data
are presented in Figure 3 and a one-way analysis of the
variance did not show any significant relationship between
GSH concentration and response (ANOVA, P=0.32).

Tumour GST isoenzyme expression and its relationship to
response to carboplatin

GST isoenzyme expression was assessed by immunohisto-
chemistry using polyclonal antibodies on fixed tumour
samples from the same 39 patients whose tumours were
analysed for GSH content. For each isoenzyme, the intensity
of the staining and the percentage of tumour cells stained was

I            I            I                                     I            i            I            I           I                                      I            I

.

PD      SD      PR       CR

Response to chemotherapy

Figure 2 The relationship between tumour glutathione concen-
tration and response to carboplatin in EOC, each point is an
individual patient.

I

I       I       I       I

PD     SD       PR      CR

Response to chemotherapy

Figure 3 The relationship between mean glutathione concentra-
tion and response to carboplatin in EOC (mean+s.d.).

recorded. For the purpose of this study, and in analysing the
results in relation to response, patients were divided into two
groups: responders (complete and partial) and non-respon-
ders (static or progressive disease); GST isoenzyme expression
was defined as positive if the staining intensity was strong
and more than 10% of the tumour cells stained positive.
Most of the tumour samples (34/39) had positive expression
of it GST, while only two different tumours expressed oa or i

GST. Table II summarises the results of GST isoenzyme
expression and its relation to response to carboplatin.
Analysis of this result by Fisher's exact and chi-squared
tests did not show any significant relationship between
response to carboplatin and GST isoenzyme expression
(P= 0.51 -0.553, 0.98-0.99 for Fisher's exact and chi-
squared tests respectively).

Discussion

From the results presented it is clear that, in this clinical
setting, there was no marked relationship between GSH
concentrations in primary EOC tumour samples and
subsequent response to carboplatin chemotherapy. Similarly,
there was no relationship between GST isoenzyme expression
and response.

Platinum compounds are among the most effective agents
for the treatment of patients with EOC and carboplatin has
been used as a single agent in the Northern Region (UK) for

GST isoenzyme expression and response to carboplatin
S Ghazal-Aswad et al

471
Table H GST isoenzyme expression and its relation to the response

to single agent carboplatin in EOC

Responders      Non-responders     P        P

GST          (CR + PR)        (SD+PD)        (Fisher's  (Chi-

isoenzyme   + ve    -ve      + ve     -ve     exact)  squared)
Xt          24        4       9         2      0.553   0.98
oc           2       26       -        11      0.51    0.99
u            2       26               11     0.51     0.99

the last 5 years. However, the clinical usefulness of
carboplatin is limited by intrinsic resistance at initial
treatment and subsequently by the development of acquired
resistance at relapse (Rozencwieg et al., 1990).

In numerous preclinical studies (Godwin et al., 1992;
Hamilton et al., 1985; Hill et al., 1990; Lai et al., 1989;
Leyland-Jones et al., 1991; Mistry et al., 1991; Nakagawa et
al., 1990; Saburi et al., 1989) GSH and GSTs have been
implicated as important factors in intrinsic and acquired
resistance to platinum compounds. Furthermore, a number of
clinical studies have been reported which linked increased
expression of GSTs with resistance to cytotoxic drugs in a
variety of tumours including colorectal (Mulder et al., 1995)
and lung cancer (Inoue et al., 1995), in which expression of
the it isoform was implicated, and acute lymphoblastic
leukaemia (Hall et al., 1994b) in which expression of , class
GST was associated with an increased probability of relapse.
However, it should be noted that several other mechanisms of
resistance have also been suggested from preclinical work,
including altered metallothionen content, increased DNA
repair and reduced cellular accumulation, and the relative
contribution of these various parameters to clinical resistance
is currently unclear.

In the clinical setting, Green et al. (1993) studied GST
expression in benign and malignant ovarian tumours and, in
agreement with the present study, found that it GST was the
most prevalent, being present in the majority of the
malignant epithelia and 83% of the non-malignant tissue.
There was no significant difference in the overall distribution
of positive staining for i, a or p in the malignant and non-
malignant biopsies, although the intensity of staining was
greater in malignant epithelia. There was no significant
association between survival and the presence or absence of a
or it GST subtypes, but the patients who showed resistance to
chemotherapy (oral chlorambucil, single-agent cisplatin or a
combination of cisplatin and cyclophosphamide), as opposed
to responding patients, were found to have higher staining
intensity for X GST (a Kruskal-Wallis test applied to the
data for tumour response against staining intensity gave a P-
value of 0.003).

Van der Zee (1992) studied GST activity and isoenzyme
expression in benign ovarian tumours, untreated malignant
ovarian tumours and malignant ovarian tumours after
platinum -cyclophosphamide chemotherapy. In untreated
malignant tumours GST activity and i GST expression
were not related to histological type, differentiation grade or
tumour volume index. GST isoenzyme patterns were identical
in benign tumours and in malignant tumours, both before
and after platinum-cyclophosphamide chemotherapy, with i

GST again being the predominant isoform. As in the study
reported here, no role for GSTs as a predictor of response
and hence a drug resistance mechanism was suggested. A
similar finding was reported by Murphy et al. (1992), who
studied GST activity and isoenzyme expression in human
ovarian cancer tumours both before and after cytotoxic
chemotherapy. Again, there was no statistically significant
difference between GST activity or isoenzyme distribution in
the two groups of patients. These authors observed that GST
activities in both pre-chemotherapy and post-chemotherapy
tumours were significantly higher than GST activity in
normal ovaries. Also, a isoenzyme expression was higher in
the normal ovary than in tumour tissue. In the work of

2U UUU

.

C=

O .m

0._

a)

C a)
0 >

O QO
a) 0-
C.)

C a)
0 O

F -

16 000

12 000

8000

4000

.

.

_ U

- I

l

I     U

I

I

7

I

*     I

U

U

U

a     I

0.
0

0-=

ai) V

O >c

)'a

:3 4 -

O m

> U)
C 0)

0

4 CL
C-.
4- )

20 000

16 000

12 000

8000

4000

0

I1

I1

..

,en nnn%f _

r-

_

_

_

r-

_-

_

_

_-

GST isoenzyme expression and response to carboplatin
a7^                                                    S Ghazal-Aswad et a!

A 79

Murphy et al. (1992), cytotoxic chemotherapy consisted of
five different regimes of drugs with a small number of
patients in each group, which prevented an analysis of the
relationship between GST expression and response to
therapy.

In conclusion, the results presented in this study show that
there was no significant relationship between GSH concen-
tration or GST isoenzyme expression and response to single-
agent carboplatin chemotherapy in EOC. These data do not
support the hypothesis that GSH content or GST isoenzyme
expression are major determinants of sensitivity to carbopla-
tin chemotherapy in primary ovarian cancer. It is important
to stress the point that this study only looked at the role of
GSH/GSTs in intrinsic resistance. The possibility still exists
that when resistance develops subsequent to chemotherapy
(acquired resistance), GSH/GSTs may play an important

References

BIAGLOW JE AND TUTTLE SW. (1993). The role of glutathione and

associated enzymes in the cellular response to radiation, peroxide
and hydroperoxides. In Drug Resistance in Oncology, Teicher BA
(ed.) pp. 309- 337. Marcel Dekker: New York.

BOLTON MG, COLVIN OM AND HILTON J. (1991). Specificity of

isozymes of murine hepatic glutathione S-transferase for the
conjugation of glutathione with L-phenylalanine mustard. Cancer
Res., 51, 2410-2415.

BURN J. (1979). Immunohistochemical methods and their applica-

tion in routine laboratory. In Recent Advances in Histopathology
10. Anthony PP and MacSween RNW. (eds) pp. 337-350.
Churchill Livingstone: Edinburgh.

CALVERT AH, NEWELL DR, GUMBRELL LA, O-REILLY S,

BURNELL N, BOXALL FE, SIDDIK ZH, JUDSON IR, GORE ME
AND WILTSHAW E. (1989). Carboplatin dosage: prospective
evaluation of a simple formula based on renal function. J. Clin.
Oncol., 11, 1748-1756.

CIACCIO PJ AND TEW KD. (1993). Glutathione S-Transferases. In

Drug Resistance in Oncology. Teicher BA. (ed.) pp. 351-374.
Marcel Dekker: New York.

COTGREAVE IA AND MOLDEUS P. (1986). Methodologies for the

application of monobromobimane to the simultaneous analysis of
soluble and protein thiol components of biological systems. J.
Biochem. Biophys. Methods, 13, 231-249.

DE GRAEFF A, SLEBOS RJC AND RODENHUIS S. (1988). Resistance

to cisplatin and analogues: mechanisms and potential clinical
implications. Cancer Chemother. Pharmacol., 22, 325-332.

GODWIN AK, MEISTER A, O'DWYER PJ, HUANG CS, HAMILTON CT

AND ANDERSON ME. (1992). High resistance to cisplatin in
human ovarian cancer cell lines is associated with marked increase
of glutathione synthesis. Proc. Natl Acad. Sci. USA, 89, 3070-
3074.

GREEN JA, ROBERTSON LJ AND CLARK AH. (1993). Glutathione S-

transferase expression in benign and malignant ovarian tumours.
Br. J. Cancer, 68, 235-239.

GULICK AM AND FAHL WE. (1995). Mammalian glutathione S-

transferase: regulation of an enzyme system to achieve
chemotherapeutic efficacy. Pharmacol. Ther., 66, 237 - 257.

HALL A, FOSTERS S, PROCTOR SJ AND CATTAN AR. (1990).

Purification and characterization of i class glutathione S-
transferase from human leukemia cells. Br. J. Haematol., 76,
494- 500.

HALL AG, MATHESON E, HICKSON ID, FOSTER SA AND HOGARTH

L. (1994a). Purification of an a class glutathione S-transferase
from melphalan-resistant Chinese hamster ovary cells and
demonstration of its ability to catalyze melphalan-glutathione
adduct formation. Cancer Res., 54, 3369-3372.

HALL AG, AUTZEN P, CATTAN AR, MALCOLM AJ, COLE M,

KERNAHAN J AND REID MM. (1994b). Expression of ju class
glutathione S-transferase correlates with event-free survival in
childhood acute lymphoblastic leukemia. Cancer Res., 54, 5251 -
5254.

HAMILTON TC, WINKER MA, LOUIE KG, BATIST G, BEHRENS BC,

TSURUO T, GROTZINGER KR, MCKOY WM, YOUNG RC AND
OZOLS RF. (1985). Augmentation of adriamycin, melphalan and
cisplatin cytotoxicity in drug-resistant and -sensitive human
ovarian carcinoma cell lines by buthionine sulfoximine mediated
glutathione depletion. Biochem. Pharmacol., 34, 2583-2586.

role. However, this issue is difficult to address clinically as the
only way to do so is by sequential biopsy before and after
chemotherapy, which is not always practical in a routine
clinical setting.

Acknowledgements

The authors would like to thank and acknowledge the financial
support of the North of England Cancer Research Campaign,
Northern and Yorkshire Regional Health Authority Research
Committee, the Leukaemia Research Fund and the North of
England Children's Cancer Research Fund. The help of research
nurses and data managers at Newcastle General Hospital, and Mrs
MR Walker in the pathology department at Queen Elizabeth
Hospital in performing this research is also gratefully acknowl-
edged.

HARRISON DJ, KHARBANDA R, CUNNINGHAM DS, MCLELLAN LI

AND HAYS JD. (1989). Distribution of glutathione S-transferase
isoenzymes in human kidney: basis for possible markers for renal
injury. J. Clin. Pathol., 42, 624-628.

HAYES PC, HARRISON DJ, BOUCHIER IAD, MCLELLAN LI AND

HAYES JD. (1989). Cytosolic and microsomal glutathione S-
transferase isoenzymes in normal human liver and intestinal
epithelium. Gut, 30, 854-859.

HILL BT, SHELLARD SA, HOSKING LK, FICHTINGER-SCHEPMAN

AMJ AND BEDFORD P. (1990). Enhanced DNA repair and
tolerance of DNA damage associated with resistance to cis-
diamminedichloroplatinum(II) after in vitro exposure of a human
teratoma cell line to fractionated X-irradiation. Int. J. Radiat.
Oncol. Biol. Phys., 19, 75-83.

INOUE T, ISHIDA T, SUGIO K, MAEHARA Y AND SUGIMACHI K.

(1995). Glutathione S transferase Pi is a powerful indicator in
chemotherapy of human lung squamous-cell carcinoma. Respira-
tion, 62, 223-227.

ISHIKAWA T AND ALI-OSMAN F. (1993). Glutathione-associated

cis-diamminedichloroplatinum(II) metabolism and ATP-depen-
dent efflux from leukemia cells. Molecular characterization of
glutathione-platinum complex and its biological significance. J.
Biol. Chem., 268, 20116-20125.

ISHIKAWA T, WRIGHT CD AND ISHIZUKA H. (1994). GS-X pump is

functionally over expressed in cis-diamminedichloroplatinum(II)-
resistant human leukemia HL-60 cells and down-regulated by cell
differentiation. J. Biol. Chem., 269, 29085-29093.

LAI GM, OZOLS RF, YOUNG RC AND HAMILTON TC. (1989). Effect

of glutathione on DNA repair in cisplatin-resistant human
ovarian cancer cell lines. J. Natl Cancer Inst., 81, 535-539.

LEYLAND-JONES BR, TOWNSEND AJ, TU CD, COWAN KC AND

GOLDSMITH ME. (1991). Antineoplastic drug sensitivity of
human MCF-7 breast cancer cell stably transfected with a
human a class glutathione S-transferases gene. Cancer Res., 51,
587- 594.

MANTLE T, MCCUSKER FM, PHILLIPS M AND BOYCE S. (1990).

Glutathione S-transferases. Isoenzymes, 18, 175- 177.

MEYER DJ, GILMORE KS, HARRIS JM, HARTLEY JA AND

KETTERER B. (1992). Chlorambucil-monoglutathionyl conju-
gate is sequestered by human alpha class glutathione S-
transferases. Br. J. Cancer, 66, 433-438.

MISTRY P, KELLAND LR, ABEL G, SIDHAR S AND HARRAP KR.

(1991). The relationships between glutathione, glutathione S-
transferase and cytotoxicity of platinum drugs and melphalan in
eight human ovarian carcinoma cell lines. Br. J. Cancer, 64, 215 -
220.

MULDER TPJ, VERSPAGET HW, SIER CFM, ROELOFS HMJ,

GANESH S, GRIFFIOEN G AND PETERS WHM. (1995).
Glutathion S-transferase ir in colorectal tumors is predictive for
overall survival. Cancer Res., 55, 2696-2702.

MURPHY D, MCGOWN AT, HALL A, CATTAN A, CROWTHER D

AND FOX BW. (1992). Glutathione S-transferase activity and
isoenzyme distribution in ovarian tumour biopsies taken before
or after cytotoxic chemotherapy. Br. J. Cancer, 66, 937-942.

NAKAGAWA K, SAIJO N, TSUCHIDA 5, SAKAI M, TSUNOKAWA Y

AND TEW KD. ( 1990). Glutathione-S-transferase Xt as a
determinant of drug resistance in transfected cell lines. J. Biol.
Chem., 265, 4296 -4301.

References

BIAGLOW JE AND TUTTLE SW. (1993). The role of glutathione and

associated enzymes in the cellular response to radiation, peroxide
and hydroperoxides. In Drug Resistance in Oncology, Teicher BA
(ed.) pp. 309- 337. Marcel Dekker: New York.

BOLTON MG, COLVIN OM AND HILTON J. (1991). Specificity of

isozymes of murine hepatic glutathione S-transferase for the
conjugation of glutathione with L-phenylalanine mustard. Cancer
Res., 51, 2410-2415.

BURN J. (1979). Immunohistochemical methods and their applica-

tion in routine laboratory. In Recent Advances in Histopathology
10. Anthony PP and MacSween RNW. (eds) pp. 337-350.
Churchill Livingstone: Edinburgh.

CALVERT AH, NEWELL DR, GUMBRELL LA, O-REILLY S,

BURNELL N, BOXALL FE, SIDDIK ZH, JUDSON IR, GORE ME
AND WILTSHAW E. (1989). Carboplatin dosage: prospective
evaluation of a simple formula based on renal function. J. Clin.
Oncol., 11, 1748-1756.

CIACCIO PJ AND TEW KD. (1993). Glutathione S-Transferases. In

Drug Resistance in Oncology. Teicher BA. (ed.) pp. 351-374.
Marcel Dekker: New York.

COTGREAVE IA AND MOLDEUS P. (1986). Methodologies for the

application of monobromobimane to the simultaneous analysis of
soluble and protein thiol components of biological systems. J.
Biochem. Biophys. Methods, 13, 231-249.

DE GRAEFF A, SLEBOS RJC AND RODENHUIS S. (1988). Resistance

to cisplatin and analogues: mechanisms and potential clinical
implications. Cancer Chemother. Pharmacol., 22, 325-332.

GODWIN AK, MEISTER A, O'DWYER PJ, HUANG CS, HAMILTON CT

AND ANDERSON ME. (1992). High resistance to cisplatin in
human ovarian cancer cell lines is associated with marked increase
of glutathione synthesis. Proc. Natl Acad. Sci. USA, 89, 3070-
3074.

GREEN JA, ROBERTSON LJ AND CLARK AH. (1993). Glutathione S-

transferase expression in benign and malignant ovarian tumours.
Br. J. Cancer, 68, 235-239.

GULICK AM AND FAHL WE. (1995). Mammalian glutathione S-

transferase: regulation of an enzyme system to achieve
chemotherapeutic efficacy. Pharmacol. Ther., 66, 237 - 257.

HALL A, FOSTERS S, PROCTOR SJ AND CATTAN AR. (1990).

Purification and characterization of i class glutathione S-
transferase from human leukemia cells. Br. J. Haematol., 76,
494- 500.

HALL AG, MATHESON E, HICKSON ID, FOSTER SA AND HOGARTH

L. (1994a). Purification of an a class glutathione S-transferase
from melphalan-resistant Chinese hamster ovary cells and
demonstration of its ability to catalyze melphalan-glutathione
adduct formation. Cancer Res., 54, 3369-3372.

HALL AG, AUTZEN P, CATTAN AR, MALCOLM AJ, COLE M,

KERNAHAN J AND REID MM. (1994b). Expression of ju class
glutathione S-transferase correlates with event-free survival in
childhood acute lymphoblastic leukemia. Cancer Res., 54, 5251 -
5254.

HAMILTON TC, WINKER MA, LOUIE KG, BATIST G, BEHRENS BC,

TSURUO T, GROTZINGER KR, McKOY WM, YOUNG RC AND
OZOLS RF. (1985). Augmentation of adriamycin, melphalan and
cisplatin cytotoxicity in drug-resistant and -sensitive human
ovarian carcinoma cell lines by buthionine sulfoximine mediated
glutathione depletion. Biochem. Pharmacol., 34, 2583-2586.

HARRISON DJ, KHARBANDA R, CUNNINGHAM DS, MCLELLAN LI

AND HAYS JD. (1989). Distribution of glutathione S-transferase
isoenzymes in human kidney: basis for possible markers for renal
injury. J. Clin. Pathol., 42, 624-628.

HAYES PC, HARRISON DJ, BOUCHIER IAD, MCLELLAN LI AND

HAYES JD. (1989). Cytosolic and microsomal glutathione S-
transferase isoenzymes in normal human liver and intestinal
epithelium. Gut, 30, 854-859.

HILL BT, SHELLARD SA, HOSKING LK, FICHTINGER-SCHEPMAN

AMJ AND BEDFORD P. (1990). Enhanced DNA repair and
tolerance of DNA damage associated with resistance to cis-
diamminedichloroplatinum(II) after in vitro exposure of a human
teratoma cell line to fractionated X-irradiation. Int. J. Radiat.
Oncol. Biol. Phys., 19, 75-83.

INOUE T, ISHIDA T, SUGIO K, MAEHARA Y AND SUGIMACHI K.

(1995). Glutathione S transferase Pi is a powerful indicator in
chemotherapy of human lung squamous-cell carcinoma. Respira-
tion, 62, 223-227.

ISHIKAWA T AND ALI-OSMAN F. (1993). Glutathione-associated

cis-diamminedichloroplatinum(II) metabolism and ATP-depen-
dent efflux from leukemia cells. Molecular characterization of
glutathione-platinum complex and its biological significance. J.
Biol. Chem., 268, 20116-20125.

ISHIKAWA T, WRIGHT CD AND ISHIZUKA H. (1994). GS-X pump is

functionally over expressed in cis-diamminedichloroplatinum(II)-
resistant human leukemia HL-60 cells and down-regulated by cell
differentiation. J. Biol. Chem., 269, 29085-29093.

LAI GM, OZOLS RF, YOUNG RC AND HAMILTON TC. (1989). Effect

of glutathione on DNA repair in cisplatin-resistant human
ovarian cancer cell lines. J. Natl Cancer Inst., 81, 535-539.

LEYLAND-JONES BR, TOWNSEND AJ, TU CD, COWAN KC AND

GOLDSMITH ME. (1991). Antineoplastic drug sensitivity of
human MCF-7 breast cancer cell stably transfected with a
human a class glutathione S-transferases gene. Cancer Res., 51,
587- 594.

MANTLE T, MCCUSKER FM, PHILLIPS M AND BOYCE S. (1990).

Glutathione S-transferases. Isoenzymes, 18, 175- 177.

MEYER DJ, GILMORE KS, HARRIS JM, HARTLEY JA AND

KETTERER B. (1992). Chlorambucil-monoglutathionyl conju-
gate is sequestered by human alpha class glutathione S-
transferases. Br. J. Cancer, 66, 433-438.

MISTRY P, KELLAND LR, ABEL G, SIDHAR S AND HARRAP KR.

(1991). The relationships between glutathione, glutathione S-
transferase and cytotoxicity of platinum drugs and melphalan in
eight human ovarian carcinoma cell lines. Br. J. Cancer, 64, 215 -
220.

MULDER TPJ, VERSPAGET HW, SIER CFM, ROELOFS HMJ,

GANESH S, GRIFFIOEN G AND PETERS WHM. (1995).
Glutathion S-transferase ir in colorectal tumors is predictive for
overall survival. Cancer Res., 55, 2696-2702.

MURPHY D, MCGOWN AT, HALL A, CATTAN A, CROWTHER D

AND FOX BW. (1992). Glutathione S-transferase activity and
isoenzyme distribution in ovarian tumour biopsies taken before
or after cytotoxic chemotherapy. Br. J. Cancer, 66, 937-942.

NAKAGAWA K, SAIJO N, TSUCHIDA S, SAKAI M, TSUNOKAWA Y

AND TEW KD. (1990). Glutathione-S-transferase X as a
determinant of drug resistance in transfected cell lines. J. Biol.
Chem., 265, 4296-4301.

GST isoenzyme expression and response to carboplatin

S Ghazal-Aswad et at                                                  4

473

RANDALL BJ, ANGUS B, AKIBA R, HALL A, CATTAN AR, PROCTOR

SJ, JONES RA AND HORNE CHW. (1990). Glutathione S-
transferase (placental) as a marker of transformation in the
human cervix uteri: an immunohistochemical study. Br. J.
Cancer, 62, 614-618.

ROZENCWEIG M, MARTIN A, BELTANGADY M, BRAGMAN K,

GOODLOW J, WILTSHAW E, CALVERT H, MANGIONI C,
PECORELLI S, BOLIS G, ROCKER I, ADAMS M AND CANETTA
R. (1990). Randomised trials of carboplatin versus cisplatin in
advanced ovarian cancer. In Carboplatin (JM8) Current
Perspectives and Future Directions, Bunn PA, Canetta R, Ozols
RF and Rozencweig M (eds). pp. 175-186. W.B. Saunders:
Philadelphia.

SUBURI Y, NAKAGAWA M, ONO M, SAKAI M, MURAMATSU M,

KOHNO K AND KUMANO M. (1989). Increased expression of
glutathione S-transferase gene in cis-diamminedichloroplatinum
(II)-resistant variants of a Chinese hamster ovary cell line. Cancer
Res., 49, 7020-7025.

VAN DER ZEE AGJ, VAN OMMEN B, MEIJER C, HOLLEMA H, VAN

BALDEREN PJ AND DE VRIES EGE. (1992). Glutathione S-
transferase activity and isoenzyme composition in benign ovarian
tumours, untreated malignant ovarian tumours and malignant
ovarian tumours after platinum/cyclophosphamide chemother-
apy. Br. J. Cancer, 66, 930-936.

				


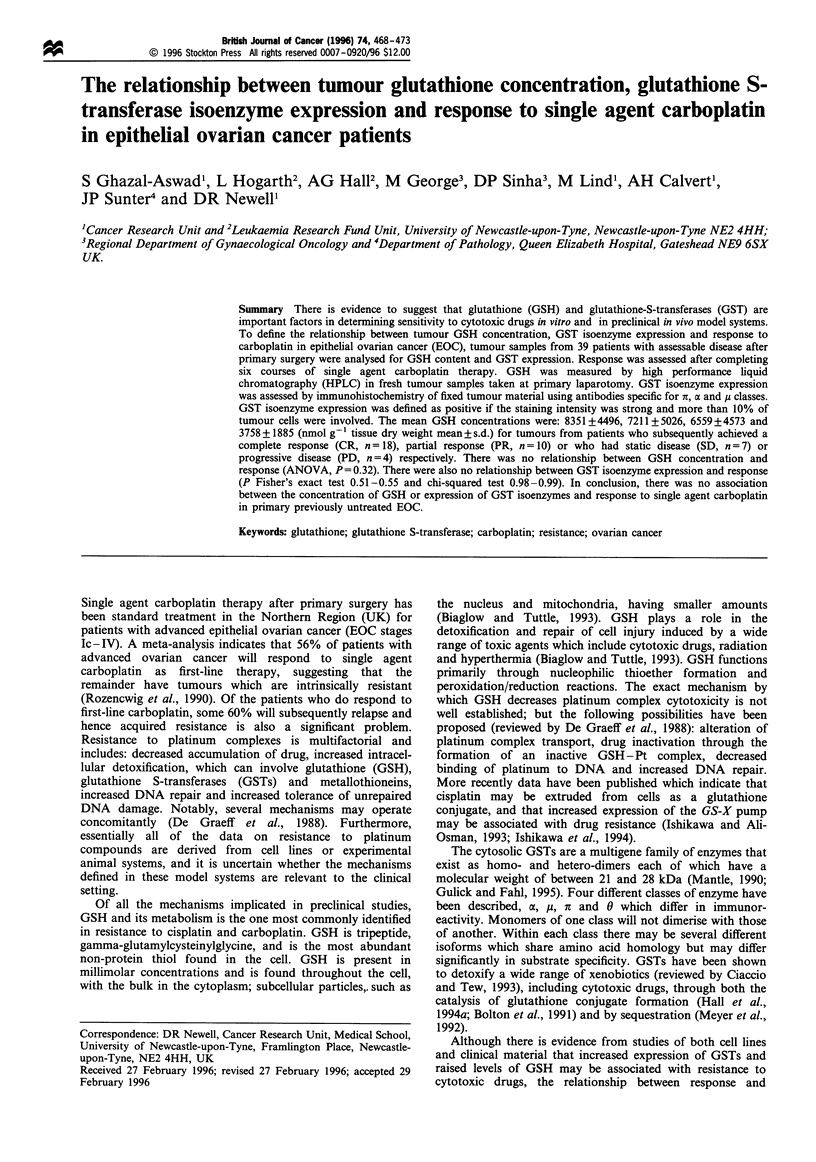

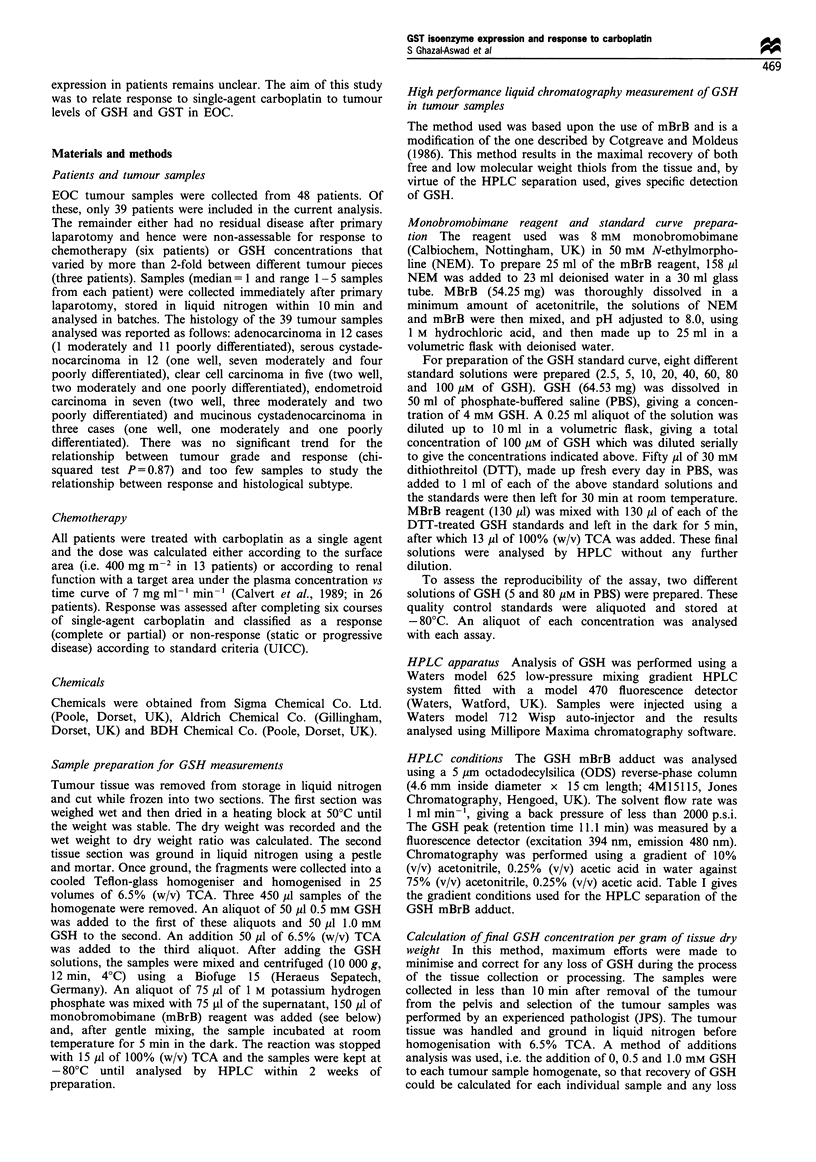

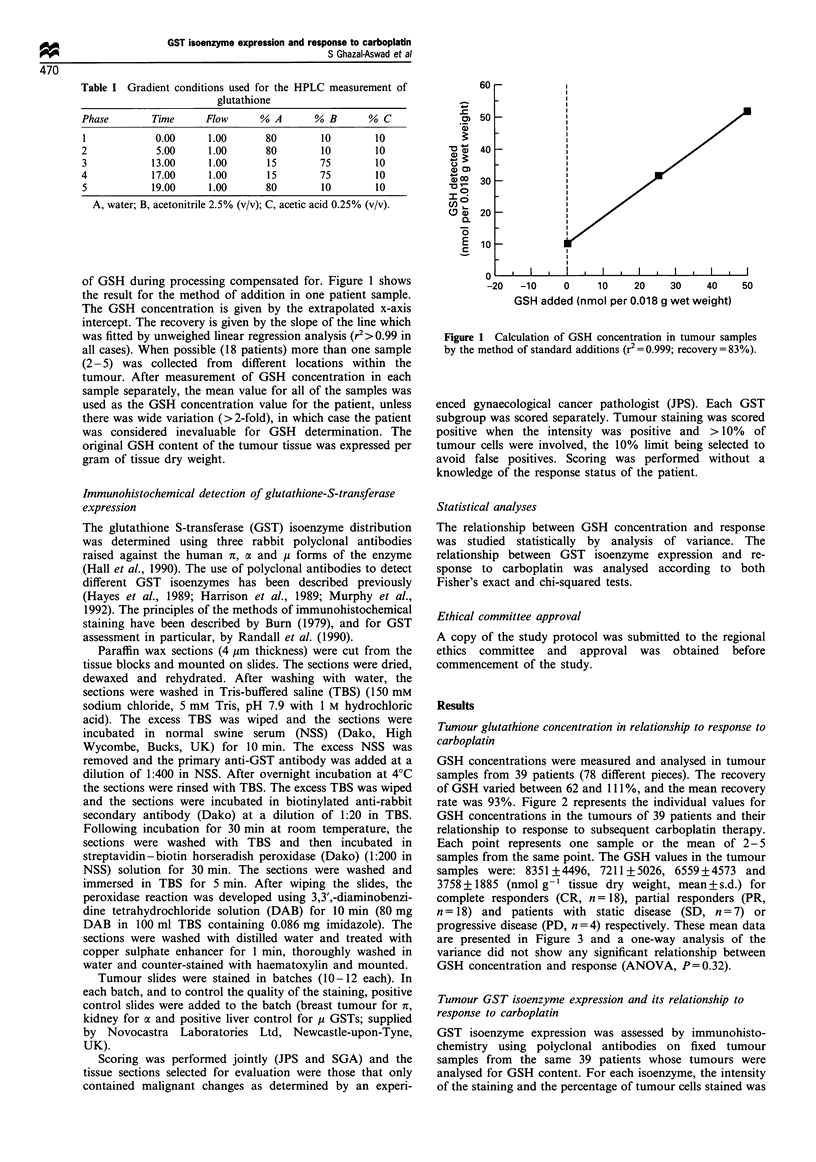

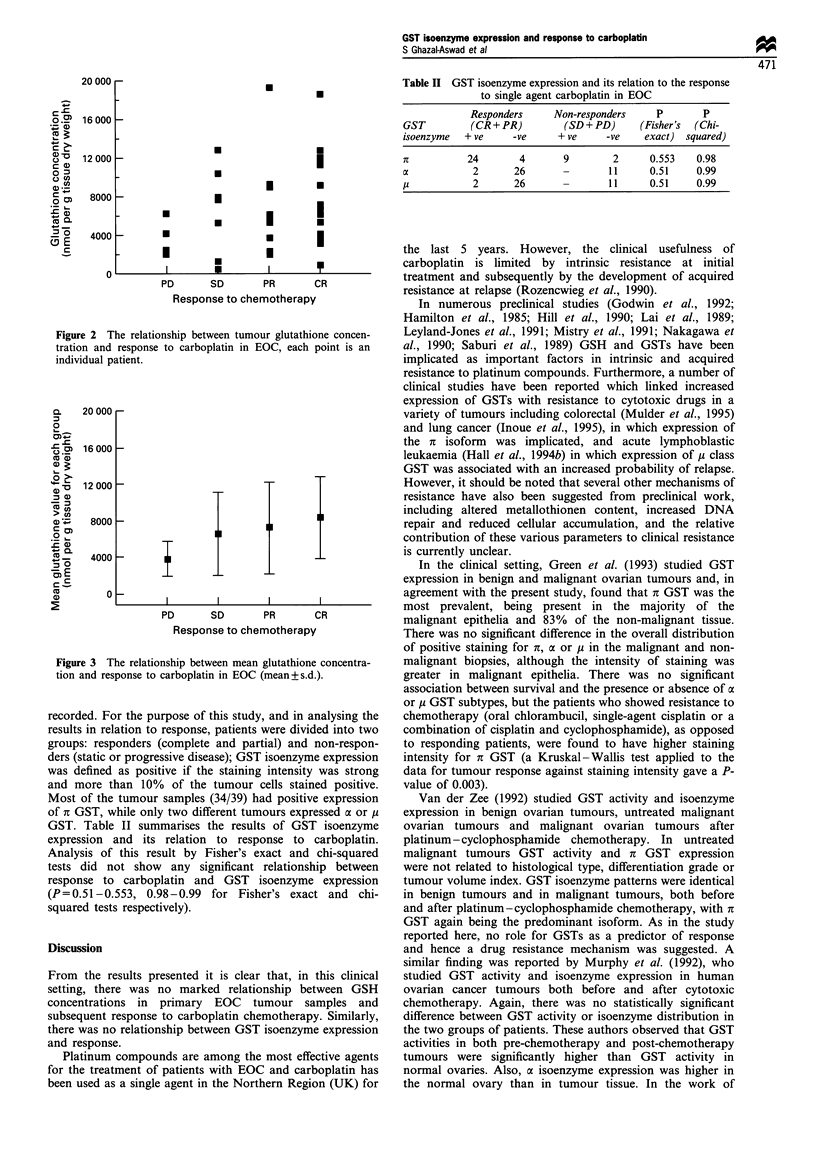

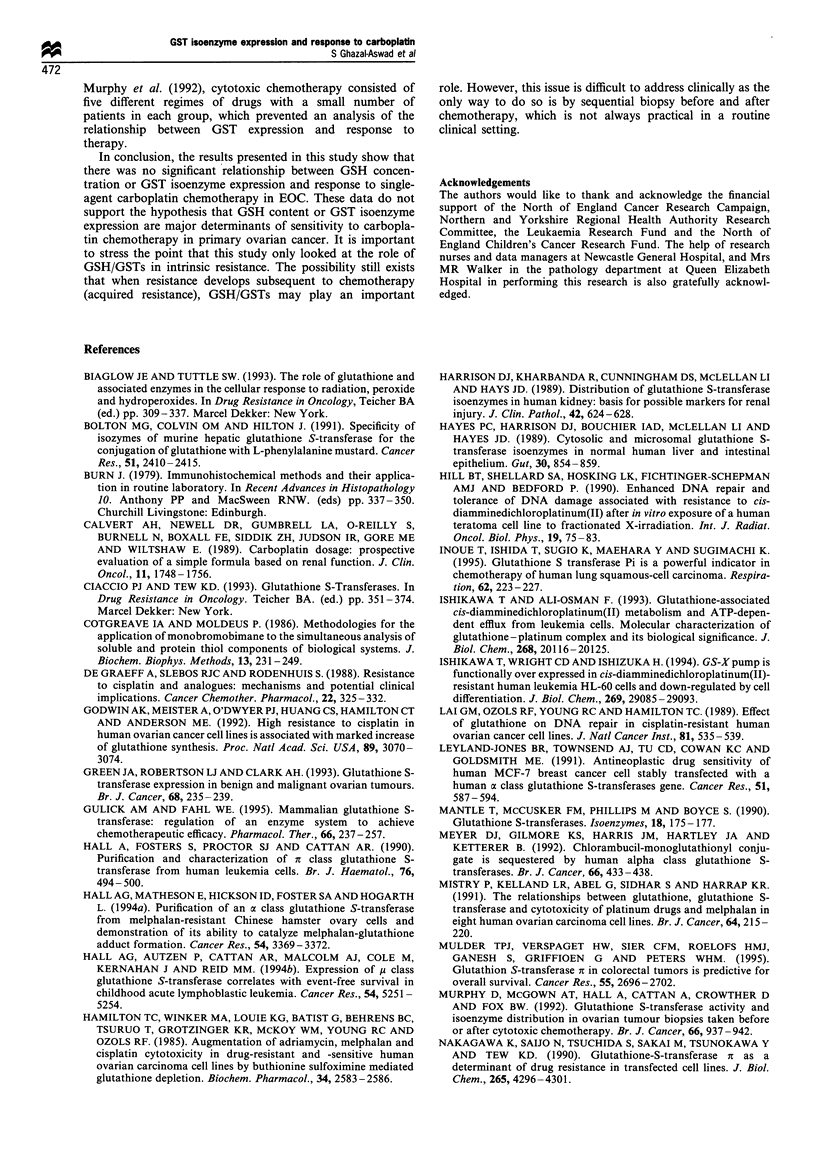

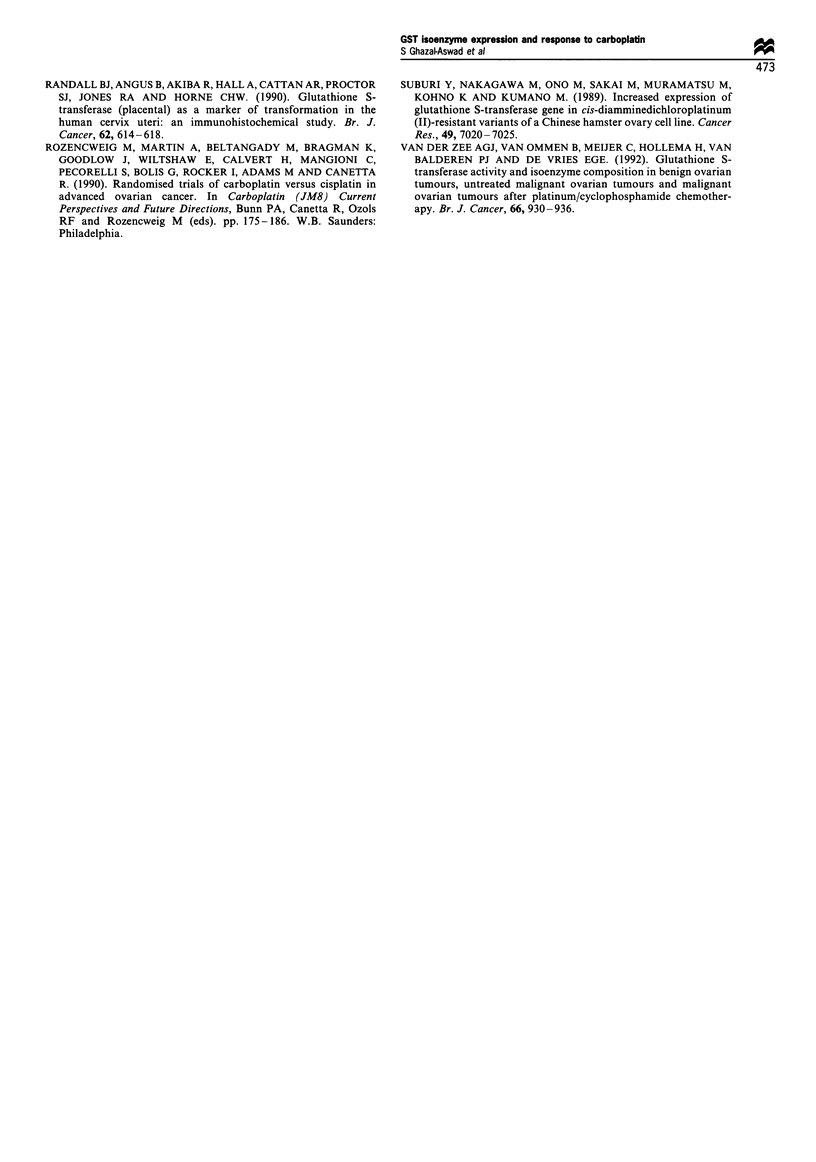

